# Clinical, Operational, and Socioeconomic Analysis of EMS Bypass of the Closest Facility for Pediatric Asthma Patients

**DOI:** 10.5811/westjem.2021.4.50382

**Published:** 2021-07-15

**Authors:** Erik Finlay, Sam Palmer, Benjamin Abes, Benjamin Abo, Jennifer N. Fishe

**Affiliations:** * University of Florida Geoplan Center, Gainesville, Florida; † Lee County Emergency Medical Services, Fort Myers, Florida; ‡ University of Florida College of Medicine – Jacksonville, Pediatric Emergency Department, Jacksonville, Florida

## Abstract

**Introduction:**

Pediatric hospital care is becoming increasingly regionalized, with fewer facilities providing inpatient care for common conditions such as asthma. That trend has major implications for emergency medical services (EMS) medical care and operations because EMS historically transports patients to the closest facility. This study describes EMS transport patterns of pediatric asthma patients in greater depth, including an analysis of facility bypass rates and the association of bypass with demographics and clinical outcomes.

**Methods:**

This was a retrospective study of pediatric asthma patients ages 2–18 years transported by Lee County, FL EMS between March 1, 2018 – December 31, 2019. A priori, we defined bypass as greater than five minutes extra transport time. We performed geospatial analysis and mapping of EMS pediatric asthma encounters. We used the Pediatric Destination Tree (PDTree) project’s tiered approach to characterize receiving hospital facility pediatric capability. We analyzed incidence and characteristics of bypass, and bypass and non-bypass patient characteristics including demographics, emergency department (ED) clinical outcomes, and socioeconomic disadvantage (SED).

**Results:**

From the study period, there were a total of 262 encounters meeting inclusion criteria, 254 (96.9%) of which could be geocoded to EMS incident and destination locations. Most encounters (72.8%) bypassed at least one facility, and the average number of facilities bypassed per encounter was 1.52. For all 185 bypass encounters, there was a median additional travel time of 13.5 minutes (interquartile range 7.5 – 17.5). Using the PDTree’s classification of pediatric capability of destination facilities, 172 of the 185 bypasses (93%) went to a Level I facility. Bypass incidence varied significantly by age, but not by minority status, asthma severity, or by the area deprivation index of the patient’s home address. Overall, the highest concentrations of EMS incidents tended to occur in areas of greater SED. With regard to ED outcomes, ED length of stay did not vary between bypass and non-bypass patients (P = 0.54), and neither did hospitalization (P = 0.80).

**Conclusion:**

We found high rates of bypass for pediatric EMS encounters for asthma exacerbations, and that bypass frequency was significantly higher in younger age groups. With national trends pointing toward increasing pediatric healthcare regionalization, bypass has significant implications for EMS operations.

## INTRODUCTION

Pediatric hospital care is becoming increasingly regionalized, with fewer facilities capable of providing inpatient care for common childhood conditions such as asthma.^1^ That trend has major implications for emergency medical services (EMS) medical care and operations because EMS historically transports patients to the closest facility. Additionally, pediatric regionalization has implications for children and their families as inpatient pediatric care may be farther away from medical homes and family support systems, especially for families of low socioeconomic status.^2^ Thus, EMS transport of pediatric patients directly to definitive care may now involve bypassing the closest facility.

A previous study in urban, suburban, and rural agencies in Maryland found that EMS bypassed the closest facility in nearly 50% of pediatric encounters.^3^ In that study, medications for asthma exacerbations (eg, bronchodilators, oxygen, and systemic corticosteroids) comprised three of the top five medications given to bypass patients.^3^ A statewide study in Florida found that the EMS provider’s destination decision was patient/family choice in nearly one-third of pediatric encounters (as opposed to closest facility), and that for 60% of patients with respiratory distress, provider destination decision was something other than the closest facility.^4^ Another Florida study analyzed the average distance for EMS to directly travel to a hospital that currently admits children for asthma exacerbations, and found average transport distances of 30 miles or greater for 11 counties.^5^

Because asthma is a common cause of pediatric emergency care^6^ and disproportionately affects minority and rural children,^7^ we sought to describe EMS transport patterns of pediatric asthma patients in greater depth. This study describes EMS bypass rates specifically for pediatric asthma exacerbations and whether bypass resulted in transport to facilities with greater pediatric care capability, and compares demographics, socioeconomic disadvantage (SED), and clinical outcomes of bypass and non-bypass patients.

## METHODS

### Study Setting, Inclusion and Exclusion Criteria

This was a retrospective study of pediatric asthma patients transported by Lee County, FL EMS between March 1, 2018 – December 31, 2019. The study was approved by the University of Florida Institutional Review Board. We obtained emergency department (ED) outcome data by EMS via their usual pediatric quality review processes. Of note, Lee County EMS asthma and respiratory distress standard operating protocols suggest that EMS providers who suspect the patient will require admission should transport to a facility with a pediatric inpatient unit.

We included encounters if the patient was between ages 2–18 years (lower limit of age two to avoid confounding with bronchiolitis), and if the EMS provider’s primary impression was asthma. We also included encounters with primary impressions indicative of respiratory distress (eg, difficulty breathing, common cold, pneumonia, etc) if the provider secondary impression was asthma *or* if albuterol was administered (either alone or in combination with ipratropium bromide). We manually reviewed charts with the provider impression allergic reaction to distinguish between allergic reactions and asthma exacerbations, as both may involve administration of albuterol. We excluded non-transports, and patients whose EMS provider primary or secondary impression was anaphylactic/anaphylactoid reaction, congestive heart failure, or chronic obstructive pulmonary disease. When examining the relationship of facility bypass to ED outcomes, we excluded encounters where ED outcome data were not available.

### Bypass, Patient, and Facility Characterizations

A priori, we defined bypass based on EMS transport time (from the EMS scene to the receiving ED) rather than distance, as time is more relevant to EMS operations and, therefore, a more transferrable metric to compare with other agencies and studies. Based on prior studies, we defined bypass as greater than five minutes extra transport time.^8^,^9^ We also performed sensitivity analyses (see Supplemental Data File) with bypass definitions of greater than three and greater than 10 minutes extra transport time.

To classify patients by asthma exacerbation severity, we used a previously published EMS pediatric asthma severity score created with elements of the 2007 National Heart, Lung, and Blood Institute’s Expert Panel Report 3 recommendations.^7^ To describe patient’s racial/ethnic background we used the race data variable from the EMS record, which combines both race and ethnicity descriptions. Therefore, we categorized patients by minority and non-minority status (Black, Asian, Hispanic/Latino, other vs White, non-Hispanic/Latino, respectively). We used the area deprivation index (ADI) to characterize SED based on the patient’s home address. The ADI is a composite measure of SED based on 17 different US Census Bureau’s (USCB) variables representing poverty, education, housing, and employment.^10^, ^11^ We used the national rank of the 2015 version ADI, which is based on demographic variables from the USCB 2011–2015 American Community Survey.^12^ We used the Pediatric Destination Tree (PDTree) project’s tiered approach to characterize receiving hospital facility pediatric capability (Level 1 – pediatric specialty center designation, Level II – pediatric intensive care unit capability, Level III – pediatric inpatient unit or separate pediatric ED, Level IV – all other facilities including freestanding EDs).^13^, ^14^

### Geospatial Analysis Methods and Area Deprivation Index Descriptions

We performed geospatial analysis and mapping of EMS pediatric asthma encounters with ArcGIS 10.5.1 (Esri, Redlands, CA). EMS scene address (incident location), destination facility address, and patient home address were geocoded using a 2018 HERE street network dataset.^15^ When home address was not available or could not be geocoded, we used the address of the EMS scene

To map neighborhood SED, ADI national rank was joined to the 2015 US Census Block groups.^16^ That information was then joined to each patient based on their home address. We categorized patients into groups based on quintiles of their ADI national rank scores. Quintile groups correspond to the following ADI scores: ADI 1 (1 – 45); ADI 2 (46 – 59), ADI 3 (60 – 77), ADI 4 (78 – 89), ADI 5 (90 – 100). The top 20^th^ percentile of ADI scores (ADI 1) represents patients with a home address in the least disadvantaged areas, while the bottom 20^th^ percentile (ADI 5) represents patients with a home address in the most disadvantaged areas.

Using the Network Analyst extension in ArcGIS, we calculated estimated transport time, in minutes, from each incident location to the actual destination facility, as well as from incident location to all other possible destination facilities within the study area. For patients who were hospitalized, we also calculated estimated travel time from patient home locations to the admitting facilities. In a supplemental analysis, we assessed the accuracy of estimated transport time by comparing it to actual transport time using simple linear regression. The supplemental figure shows a moderately strong association between estimated and actual transport time (R^2^ = 0.697). On average, transport time modeled using Network Analyst underestimated actual transport time by 3.9 (±0.7) minutes. However, the degree of underestimation remained fairly consistent across the entire range of estimates.

For each transport, we used the results of the network analysis to identify the total number of bypassed facilities along the route from incident location to the actual destination facility. If the estimated time it took to arrive at the actual facility was five minutes or greater compared to that of an alternative facility, the alternative facility was considered a bypassed location. Patient characteristics and transport/travel times were compared across bypass groups using Wilcoxon rank-sum and Fisher’s exact tests, with bypass status treated as a binary variable (no bypasses vs one or more bypasses). For analyses comparing EMS transport time as the outcome variable, we used actual recorded EMS transport time. For analyses comparing travel time from patients’ homes to admitting facilities, we used the Network Analyst-estimated travel time. For all analyses, we used descriptive statistics (mean, standard deviation [SD], median, interquartile range [IQR]) as appropriate, and univariate comparison tests (chi square for categorical variables, Wilcoxon rank-sum for continuous variables).

## RESULTS

From the study period, there were a total of 262 pediatric asthma EMS transports meeting inclusion criteria, 254 (96.9%) of which had EMS scene encounter information that we were able to geocode to incident and destination locations. Eight transports (3.1%) lacked sufficient address information at either incident location, destination location, or both, and thus could not be geocoded. For home address, 226 patients (86.3%) were geocoded. Using the five-minute definition of bypass, 72.8% of those encounters bypassed at least one facility, and the average number of facilities bypassed per encounter was 1.52. Using that five-minute bypass definition, we noted 69 incidents with 0 bypasses, 40 incidents with 1 bypass, 97 incidents with 2 bypasses, 39 incidents with 3 bypasses, and 9 incidents with 4 bypasses. Table 1 displays the incidence of bypass and descriptive statistics for number of bypasses per EMS encounter.

**Figure 1:**
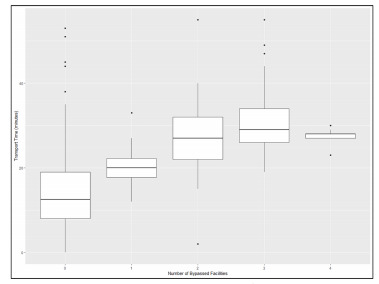
Actual transport time by number of bypasses per route. Box and whisker plots represent median (middle line), IQR (borders of box from 25th (Q1) to 75th (Q3) percentiles), edges of lines represent values within Q1 – 1.5*IQR to Q3 + 1.5*IQR; isolated dots represent outliers beyond the ±1.5*IQR values. *IQR*, interquartile range.

**Figure 2:**
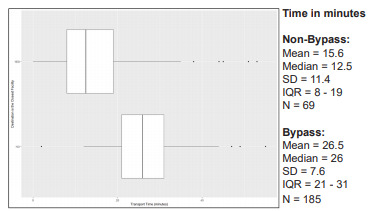
Comparison of actual transport time between bypass and non-bypass patients. Box and whisker plots represent median (middle line), IQR (borders of box from 25th (Q1) to 75th (Q3) percentiles), edges of lines represent values within (Q1 – 1.5*IQR) to (Q3 + 1.5*IQR); isolated dots represent outliers beyond the ±1.5*IQR values. *SD*, standard deviation; *IQR*, interquartile range.

Emergency medical services travel time varied greatly by number of facilities bypassed and between bypass and non-bypass patients. Figure 1 displays box and whisker plots for EMS travel time (from EMS scene to destination facility) by the number of facilities bypassed. Figure 2 shows the significantly longer EMS transport time for bypass patients compared to non-bypass patients (*P* <0.0001, Wilcoxon rank-sum test). For all 185 bypass encounters, there was a median additional travel time of 13.5 minutes (IQR 7.5 – 17.5).

**Figure 3:**
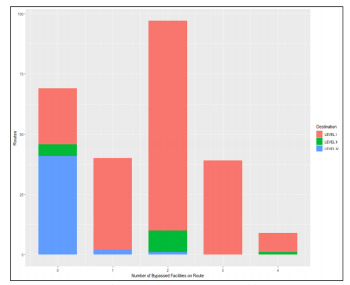
Destination facility level and number of facilities bypassed. Y axis shows number of emergency medical services encounters in the study sample; X axis shows number of facilities bypassed en route to ultimate destination. Shaded bars represent the pediatric capability of the destination facility.

Using the PDTree classification of pediatric capability of destination facilities, we found that 195 patients were transported to a Level I facility, 15 patients to a Level II facility, and four patients to a Level IV facility overall. For bypasses, 172 of the 185 bypasses (93%) went to a Level I facility, 10 went to a Level II facility, and three went to a Level IV facility. Figure 3 shows the variation in destination facility pediatric capability by number of facilities bypassed en route to that ultimate destination. For the 185 bypass encounters, the median travel time to a Level I facility was significantly longer at 26.5 minutes (IQR 22 – 32), vs all other facilities levels (median 19 minutes (IQR 17 – 23) (*P* = 0.0009, Wilcoxon rank-sum test).

**Figure 4:**
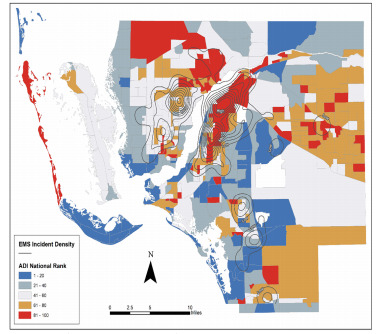
Smoothed density of emergency medical services incidents overlaid with area deprivation index quintile. *EMS*, emergency medical services; *ADI*, area deprivation index.

Examining bypass at the patient level (Table 2), we found that bypass incidence varied significantly by age but not by minority status, asthma severity, or patient’s home address ADI. Although bypass incidence did not vary by ADI or by asthma severity, there was a higher incidence of severe/critical asthma encounters in higher ADI categories (ie, more disadvantaged neighborhood groups). When breaking down ADI into quintiles, 63.5% of the fifth quintile patients were rated as severe or critical, compared to 38.8% in the first quintile (*P* = 0.01). Figure 4 shows the spatial distribution of EMS incidents in relation to ADI within Lee County.^17^ Overall, the highest concentrations of EMS incidents tended to occur in areas of greater SED.

**Figure 5:**
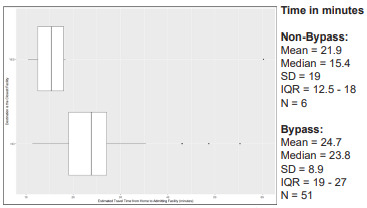
Estimated travel time from home to facility among admitted patients with home address (N = 57). Box and whisker plots represent median (middle line), IQR (borders of box from 25th (Q1) to 75th (Q3) percentiles), edges of lines represent values within Q1–1.5*IQR to Q3+1.5*IQR; isolated dots represent outliers beyond the ±1.5*IQR values. *SD*, standard deviation; *IQR*, interquartile range.

Emergency department outcomes were available for 189 of the 254 total geocoded patients. Of those 189 patients, 166 (87.8%) were bypasses, and 58 (30.7%) were admitted to the hospital. Length of stay in the ED did not vary between bypass and non-bypass patients (*P* = 0.54), and neither did hospitalization (*P* = 0.80). After geocoding the admitted patient’s home address, we used Network Analyst to calculate estimated travel time from home to the admitting facility, as bypass to a facility farther away may strain family resources. Figure 5 displays how travel time from home to admitting facility was significantly longer for bypass patients vs non-bypass patients (*P* = 0.04, Wilcoxon rank-sum test).

## DISCUSSION

In this prehospital study of asthma exacerbations, one of the most common pediatric emergency conditions, we found nearly three-quarters of EMS encounters bypassed the nearest facility, and 93% of those bypasses were to go to a Level I pediatric specialty facility. Those bypass transports included not only passing one facility, but in some cases, bypassing up to four other facilities. This study’s 72.8% overall rate of bypass is more frequent than a study of three counties in Maryland, which found an overall 50% rate of bypass when studying rural, suburban, and urban counties. This study’s high rate of bypass may reflect increasing pediatric inpatient care regionalization for asthma (despite its commonality) since that Maryland study,^1^ and/or family preference for transport to a children’s hospital in a study setting where there is one Level I pediatric facility option.^4^ Since the study EMS agency’s guidelines recommend transport to a facility with a pediatric inpatient unit if the need for admission is suspected, the bypass rates may also reflect EMS provider’s impressions of the likelihood of admission. However, bypass did not vary by asthma severity, and only 30% of bypass patients transported to the Level I facility were admitted.

The choice to transport to a higher level of pediatric facility also did not vary by SED, as represented by ADI or minority status. Interestingly, our lack of variance by SED is in contrast to a similar study of bypass from Baltimore City, which found bypass rates did vary by census tract median poverty levels.^2^ However, we did find that the highest concentrations of EMS incidents occurred in areas of the greatest SED, which is in keeping with many other studies.^18^ Therefore, the SED results and bypass rates overall may reflect an increased number of emergency destination options (Level I to IV) available for EMS transport, and this should be considered when applying our results to other areas.

Bypass did vary by patient age, with younger infants and toddlers more likely to experience bypass encounters than teenagers. That variation in age has similarly been found in studies of increasing pediatric interfacility transfers,^1^ as well as in studies of pediatric secondary transport (interfacility transport following primary EMS transport).^19^ More comprehensive studies of bypass, to include other pediatric conditions, are required to determine whether younger age is the main factor driving bypass, or if other factors related to the patient’s condition or parental preference contribute as well. Additionally, in-depth qualitative studies with EMS providers may be required to ascertain whether anchoring bias (eg, dispatch call for pediatric patient with difficulty breathing) or treatment bias (eg, being able to tell caregivers transport will be to a pediatric specialty facility) plays a role in bypass for pediatric prehospital asthma patients.

Regardless of the reason(s) for bypass, its frequency has major implications for EMS operations. We found significantly longer transport times for bypass patients with a median increase of 13.5 minutes. An extra 13.5 minutes to a layperson may not sound significant. However, a statewide study of pediatric EMS transports in Florida found an overall median transport time of 13 minutes.^4^ Therefore, bypass in this study doubled that transport time. Because EMS operates as a public service, ambulance and crew availability must be optimized for all citizens. Thus, additional travel time to definitive care must be balanced against potential additional turnaround time at specialty facilities and the further distance/time required to travel back to the ambulance’s home station. In fact, turnaround time can be significant (ranging from minutes to nearly an hour), and varies greatly between receiving facilities.^20^ Additionally, ambulance availability is a critical component to time-sensitive care for other emergencies such as stroke, trauma, ST-elevation myocardial infarction, and other medical emergencies.^21^–^23^

Aside from the public health service considerations, that additional transport time can also strain families and caregivers of pediatric asthma patients. Of the 30% of bypass patients who required admission, we found a slight but statistically significant increase in the amount of travel time from the patient’s home address to the admitting facility. Being admitted to a hospital farther from home can strain family resources when trying to visit children in the hospital while potentially caring for other children at home and/or working. Therefore, Level I pediatric facility’s social resources should be aware of this additional strain and strategize ways to help alleviate that burden.

## LIMITATIONS

This study has limitations to consider. It is a study of one EMS agency serving a specific region in Florida, and of pediatric asthma encounters only. As such, its results may not be generalizable to all regions (particularly those without Level I pediatric facilities) or conditions besides asthma. However, the study agency serves a large volume of pediatric encounters, and asthma is one of the most common reasons for pediatric EMS encounters,^6^ and may be representative of overall pediatric EMS trends. We used admission rate as a surrogate for the need to bypass closer facilities; however, this does not take into account any subspecialty consultations (eg, pediatric pulmonology or allergy) that may have occurred in the ED prior to discharge. However, given pediatric care regionalization, pediatric subspecialty consultations are usually only available at specialized pediatric facilities.

Additionally, we were not able to obtain ED outcomes for all patients, which may have biased results relating to admission rates and extra distance from home for admitted patients.

## CONCLUSION

This study of pediatric EMS encounters for asthma exacerbations found a high rate of bypass to a Level I pediatric facility, and that bypass frequency was significantly higher in younger age groups. With national trends pointing toward increasing pediatric healthcare regionalization, bypass has significant implications for EMS operations, and in certain regions may strain families and caregivers of children with asthma.

**Population Health Research Capsule**

What do we already know about this issue?

*Pediatric hospital care is becoming regionalized, and in parallel, studies show emergency medical services (EMS) bypasses the closest facility for many pediatric encounters*.

What was the research question?

*What are EMS bypass rates and characteristics for pediatric asthma patients?*

What was the major finding of the study?

*Bypass was frequent (72.8%) and more likely in younger patients. Bypass transport times were 13.5 minutes longer*.

How does this improve population health?

*EMS bypass impacts ambulance availability as it increases travel and turnaround times. Public health officials should quantify local bypass patterns and determine their local impact*.

## Supplementary Information





## Figures and Tables

**Table 1:** Bypass incidence for pediatric asthma emergency medical services encounters.

Bypass threshold	Encounters with at least 1 bypass, N (%)	Total Bypassed Facilities	Bypasses per route Mean (SD)	Bypasses per route Median (IQR)
5 minutes	185 (72.8)	387	1.52 (1.15)	2 (1 – 3)
3 minutes	192 (75.6)	460	1.81 (1.32)	2 (0 – 2)
10 minutes	126 (49.6)	169	0.67 (0.77)	0 (0 – 1)

*SD*, standard deviation; *IQR*, interquartile range.

## References

[1] França UL, McManus ML (2018). Trends in regionalization of hospital care for common pediatric conditions. Pediatrics.

[2] Fishe JN, Psoter KJ, Anders JF EMS Bypass of the Closest Hospital for Pediatric Patients Varies Significantly by Socioeconomic Status.

[3] Fishe JN, Psoter KJ, Anders JF (2019). Emergency medical services bypass of the closest facility for pediatric patients. Prehosp Emerg Care.

[4] McManus K, Finlay E, Palmer S (2020). A statewide analysis of reason for EMS’ pediatric destination choice. Prehosp Emerg Care.

[5] Fishe JN, Finlay E, Palmer S (2019). A geospatial analysis of distances to hospitals that admit pediatric asthma patients. Prehosp Emerg Care.

[6] Lerner EB, Dayan PS, Brown K (2014). Characteristics of the pediatric patients treated by the Pediatric Emergency Care Applied Research Network’s affiliated EMS agencies. Prehosp Emerg Care.

[7] Fishe JN, Palmer E, Finlay E (2019). A statewide study of the epidemiology of emergency medical services’ management of pediatric asthma. Pediatr Emerg Care.

[8] Lee J, Abdel-Aty M, Cai Q (2018). Effects of emergency medical services times on traffic injury severity: a random effects ordered probit approach. Traffic Inj Prev.

[9] Bürger A, Wnent J, Bohn A (2018). The effect of ambulance response time on survival following out-of-hospital cardiac arrest. Dtsch Arztebl Int.

[10] Singh GK (2003). Area deprivation and widening inequalities in US mortality, 1969–1998. Am J Public Health.

[11] Kind AJ, Jencks S, Brock J (2014). Neighborhood socioeconomic disadvantage and 30-day rehospitalization: a retrospective cohort study. Ann Intern Med.

[12] University of Wisconsin School of Medicine Public Health (2015). 2015 Area Deprivation Index v2.0.

[13] Fratta KA, Fishe JN, Anders JF (2019). Introduction of a new EMS protocol using the Communities of Practice Educational Model. Prehosp Disaster Med.

[14] The Pediatric Destination Tree.

[15] StreetMap Premium for ArcGIS North America HERE (formerly NAVTEQ) 2019 Release 1.

[16] 2015 TIGER/Line Shapefiles.

[17] County Boundaries in Florida (with selected fields from the 2011–2015 American Community Survey).

[18] Gold DR, Wright R (2005). Population disparities in asthma. Annu Rev Public Health.

[19] Fishe JN, Psoter K, Klein BL (2018). A retrospective evaluation of risk factors for pediatric secondary transport. Prehosp Emerg Care.

[20] Vandeventer S, Studnek JR, Garrett JS (2011). The association between ambulance hospital turnaround times and patient acuity, destination hospital, and time of day. Prehosp Emerg Care.

[21] Ashburn NP, Hendley NW, Angi RM (2020). Prehospital trauma scene and transport times for pediatric and adult patients. West J Emerg Med.

[22] Centers for Disease Control and Prevention (2007). Prehospital and hospital delays after stroke onset--United States, 2005–2006. MMWR Morb Mortal Wkly Rep.

[23] Muñoz D, Roettig ML, Monk L (2012). Transport time and care processes for patients transferred with ST-segment elevation myocardial infarction: the reperfusion in acute myocardial infarction in Carolina emergency rooms experience. Circ Cardiovasc Interv.

