# Supplementary material for: Clinical, Operational, and Socioeconomic Analysis of EMS Bypass of the Closest Facility for Pediatric Asthma Patients
Source: West J Emerg Med. 2021 Jul 15;22(4):972–8. doi: 10.5811/westjem.2021.4.50382 (PMC8328167; doi:10.5811/westjem.2021.4.50382)
Supplement: Supplementary file 1 [file wjem-22-972-s001.docx]

**Supplemental Data File**

**Sensitivity Analysis for Definition of Bypass by EMS transport time**

1. EMS transport time - bypass vs. non-bypass (Figure 2 in manuscript).

| Bypass threshold | Encounters with at least 1 bypass  N (%) | Bypass – Non-bypass  (median time in minutes) | Wilcoxon Rank-Sum  p-value |
| --- | --- | --- | --- |
| 5 minutes | 185 (72.8) | 26 – 12.5 = 13.5 | 5.086e-^16^ |
| 3 minutes | 192 (75.6) | 25 – 12 = 13 | 6.111e-^15^ |
| 10 minutes | 126 (49.6) | 28 – 18 = 10 | 2.2e-^16^ |

1. EMS transport time among bypass patients - Level I vs. All others. (Figure 3).

| Bypass threshold | Encounters going to Level I  N (%) | Level I – All others  (median time in minutes) | Wilcoxon Rank-Sum  p-value |
| --- | --- | --- | --- |
| 5 minutes | 172 (93.0) | 26.5 – 19 = 7.5 | 0.0009 |
| 3 minutes | 176 (91.6) | 26 – 19 = 7 | 0.0004 |
| 10 minutes | 119 (64.3) | 28 – 23 = 5 | 0.0181 |

1. Estimated time home to facility - Bypass vs. Non-bypass

| Bypass threshold | Encounters with at least 1 bypass  N (%) | Bypass – Non-bypass  (median time in minutes) | Wilcoxon Rank-Sum  p-value |
| --- | --- | --- | --- |
| 5 minutes | 51 (89.5) | 23.8 – 15.4 = 8.4 | 0.0438 |
| 3 minutes | 54 (94.7) | 23.2 – 13.9 = 9.3 | 0.4421 |
| 10 minutes | 36 (63.2) | 23.9 – 15.7 = 8.2 | 0.0002 |

(4) Bypass vs. Non-bypass comparison of OR (95% CI); *p value < 0.05.

| **Age Group (years)** | **OR (5 min)** | **OR (3 min)** | **OR (10 min)** |
| --- | --- | --- | --- |
| 0-4 (ref) | 1 | 1 | 1 |
| 5-9 | 1.15 (0.45-2.72) | 0.63 (0.24-1.67) | 0.65 (0.32-1.32) |
| 10-14 | 1.01 (0.42-2.45) | 0.64 (0.23-1.70) | 0.95 (0.46-1.99) |
| 15-18 | 0.34 (0.16-0.77)* | 0.20 (0.08-0.48)* | 0.43 (0.21-0.88)* |
| **Race / Ethnicity** |  |  |  |
| White (ref) | 1 | 1 | 1 |
| Minority | 0.88 (0.50-1.55) | 1.03 (0.58-1.84) | 1.39 (0.84-2.30) |
| **Severity** |  |  |  |
| Mild (ref) | 1 | 1 | 1 |
| Moderate | 0.96 (0.36-2.56) | 1.01 (0.37-2.70) | 1.25 (0.51-3.11) |
| Severe / Critical | 1.19 (0.45-3.11) | 1.52 (0.58-4.05) | 1.58 (0.66-3.81) |
| **ADI Quintile** |  |  |  |
| ADI 1 (ref) | 1 | 1 | 1 |
| ADI 2 | 1.39 (0.57-3.38) | 1.41 (0.57-3.51) | 1.58 (0.72-3.50) |
| ADI 3 | 1.10 (0.46-2.61) | 1.23 (0.50-3.01) | 1.64 (0.74-3.66) |
| ADI 4 | 1.52 (0.62-3.74) | 1.55 (0.61-3.93) | 2.26 (1.01-5.05) |
| ADI 5 | 0.91 (0.39-2.09) | 0.98 (0.42-2.33) | 2.70 (1.22-6.01)* |
| **ADI percentile** |  |  |  |
| ADI (1-89) (ref) | 1 | 1 | 1 |
| ADI (90-100) | 0.74 (0.38-1.42) | 0.77 (0.39-1.53) | 1.73 (0.94-3.21) |

(5) Hospitalization - Bypass vs. non-bypass

| **Hospitalization** | **OR (5 min)** | **OR (3 min)** | **OR (10 min)** |
| --- | --- | --- | --- |
| No | 1 | 1 | 1 |
| Yes | 1.29 (0.48-3.47) | 2.55 (0.71-9.12) | 1.11 (0.59-2.10) |

(6) ED LOS - Bypass vs. non-bypass

| Bypass threshold | Encounters with at least 1 bypass  N (%) | Bypass – Non-bypass  (median ED LOS in hours) | Wilcoxon Rank-Sum  p-value |
| --- | --- | --- | --- |
| 5 minutes | 166 (87.8) | 2.81 – 2.52 = 0.29 | 0.5378 |
| 3 minutes | 170 (89.9) | 2.96 – 2.43 = 0.53 | 0.1507 |
| 10 minutes | 114 (60.3) | 2.96 – 2.81 = 0.15 | 0.8448 |
