# Supplementary material for: Clinical, Operational, and Socioeconomic Analysis of EMS Bypass of the Closest Facility for Pediatric Asthma Patients
Source: West J Emerg Med. 2021 Jul 15;22(4):972–8. doi: 10.5811/westjem.2021.4.50382 (PMC8328167; doi:10.5811/westjem.2021.4.50382)
Supplement: Supplementary file 2 [file wjem-22-972-s002.docx]

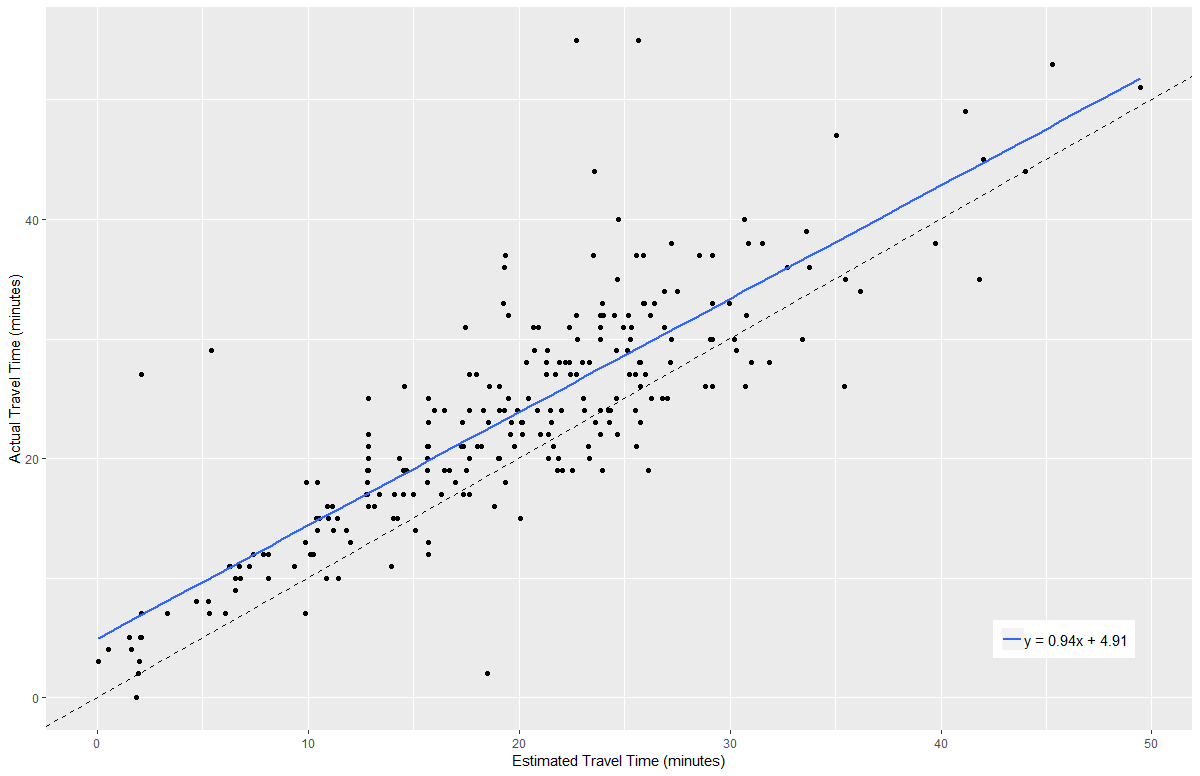


**Actual Transport Time = 4.91 + 0.94*Estimated Transport Time**

**Coefficients: Estimate Std. Error t value Pr(>|t|)**

(Intercept) 4.91084 0.84632 5.803 1.96e-08

Estimated Travel Time 0.94803 0.03932 24.112 < 2e-16

---

Multiple R-squared: 0.6985, Adjusted R-squared: 0.6973

**Results**

**Supplemental Figure.** Actual recorded EMS transport time versus ArcGIS Network Analyst estimated transport time.
